# Risodontrypy, or Hullihen’s Operation

**Published:** 1853-01

**Authors:** 


### Editorial.

### RISODONTRYPY, OR HULLIHEN'S OPERATION.

The new method of treating exposed nerves by Dr. Hullihen is exciting a good deal of interest in the profession; and we see some little *feeling* also. It appears that Dr. S. P. Miller of Worcester, Mass., has been pursuing much the same practice since June, 1850; and immediately after the meeting of the American Society of Dental Surgery last August, reported for the Bost. Med. and Sur. Journal, his method of treatment, claiming as a matter of course, originality. He at least not noticing the fact that the same operation had been performed by Dr. Hullihen, and through his recommendation by Prof. Cone and others. It is this last fact alone which looks unprofessional. Had Dr. Miller's article appeared before the meeting of the society; where this operation appears to have been discussed, and when Dr. Miller was present, it would all have been right and proper, for we have no evidence that it is not original with Dr. Miller as well as Dr. Hullihen. We believe he claims to have brought it before the American Society of Dental Surgeons through Dr. Bridges, a year previous to the time when Dr. Hullihen made it known through Dr. Cone. This would do away with much of the objection to the Dr's. silence in regard to Dr. Hullihen; for if the society treated his previous disclosure with silence, it could hardly blame Dr. Miller for the same thing. But we have next the testimonials to establish priority in this. Dr. Hullihen, has, so far as we have seen. the advantage; for he goes back to 1846, having performed the operation in the *summer or fall* of that year for Dr. J. Frissell of Wheeling, and even before this according to the testimony given by Dr Frissell in a letter to Dr. Hullihen, in which he remarks: "That from before the time you performed the first operation on me, I have, through your invitations, had frequent opportunities to witness the performance of the operation, as well as to examine cases in which the operation had previously been performed." We presume that Dr. Miller will scarce go farther back than this. This is over six years. We wait, however. for further disclosures, and in the mean time would suggest to those holding back improvements, that others may also be in the same fix; and the better way to secure priority is to give what they know to the profession, through some of the periodicals established for that purpose.

We have as yet had no opportunity of performing the operation, although we have repeatedly capped the nerve and plugged teeth since we noticed it in our last issue, and but in one instance has the operation failed in our hands in the last six months. We are favorably impressed, however, with the operation as directed and performed by Dr. Hullihen, and shall try it as soon as we can get a patient to submit, and would not hesitate to give chloroform for that purpose.

Since penning the above we have performed the operation on the anterior superior bicuspid of the right side; and succeeded in filling the tooth without any difficulty. How it may terminate we know not. The patient complained of but little pain, much less than we expected.

Dr. Cone's report of cases treated by this method are interesting and have been crowded out of this number of the Register, but will appear in the next.

